# Activation of the transcription factor, nuclear factor kappa-B, during the estrous cycle and early pregnancy in the pig

**DOI:** 10.1186/1477-7827-8-39

**Published:** 2010-04-28

**Authors:** Jason W Ross, Morgan D Ashworth, Daniel Mathew, Patrick Reagan, Jerry W Ritchey, Kanako Hayashi, Thomas E Spencer, Matthew Lucy, Rodney D Geisert

**Affiliations:** 1 Department of Animal Science, Oklahoma State University, Stillwater, OK 74078, USA; 2 Department of Animal Science, University of Missouri, Columbia, MO 65211, USA; 3 Department of Veterinary Pathobiology, Oklahoma State University, Stillwater, Oklahoma 74078, USA; 4 Department of Animal Science, Texas A&M University, College Station, TX 77843, USA; 5 Iowa State University, College of Agriculture and Life Sciences, Department of Animal Science, Ames, IA 50011, USA; 6 Department of Physiology, Southern Illinois University School of Medicine, Carbondale, IL 62901, USA; 7 University of Missouri-Columbia, College of Agriculture, Food and Natural Resources, Animal Science Division, Columbia, MO 65203, USA

## Abstract

Establishment and maintenance of pregnancy in the pig involves intricate communication between the developing conceptuses and the maternal endometrium. This process occurs during trophoblast elongation which is spaciotemporally associated with conceptus synthesis and release of IL1B concomitant with pregnancy-specific endometrial up-regulation of IL-1 receptors, providing the potential for activation of the transcription factor, NFKB. The objective of the current investigation was to determine changes in expression and cellular localization of NFKB and associated factors during the estrous cycle and early pregnancy in the pig. In situ hybridization was used to localize changes in PGR, ESR1, and TNFRSF11A during the peri-implantation period. Quantitative RT-PCR was utilized to demonstrate gene expression changes for NFKB1, RELA, TNFRSF11A, TLR4, NFKBIA and NFKBIB. Transcription factor ELISA demonstrated an overall increase in RELA during the peri-implantation period in both cyclic and pregnant gilts. While the presence of TNFSF11A and TLR4 were both detected, TLR4 expression changes were temporally associated with NFKB expression and activation. Collectively, these data demonstrate that NFKB activation may occur during the period of uterine receptivity in both the cyclic and pregnant endometrium.

## Background

Establishment of pregnancy requires changes in the uterus that allow for attachment and implantation of a developing conceptus. Given the fact that the uterine transcriptional profile during early conceptus development resembles a proinflammatory response, it is possible that the transcription factor, nuclear factor κB (NFKB), is involved in the establishment of pregnancy. NFKB consists of multiple subunits that have a common Rel homology domain [1]. Inactive NFKB is sequestered in the cytoplasm through binding to inhibitors of NFKB (IKBs) until activation by IKB kinases, which results in the release and ubiquitination of IKB and the subsequent translocation of NFKB dimers into the nucleus regulating transcription of responsive genes [1,2]. NFKB activation can be caused by numerous stimuli such as bacterial endotoxin lipopolysaccharide, reactive oxygen species, and cytokines such as tumor necrosis factor α (TNF) and interleukin-1β (IL1B). The pregnancy-specific increase of IL1B in the uterine lumen during conceptus elongation and attachment in pigs [3] may contribute to the activation of NFKB and the transcription of regulated genes, such as prostaglandin synthase-2 (PTGS2). Interestingly, PTGS2 expression is not pregnancy specific as both expression of the gene and enzyme are up-regulated in the luminal epithelium (LE) of gilts beginning on days 10-12 of the estrous cycle and early pregnancy [4]. Indomethacin, a non-steroidal anti-inflammatory drug that inhibits prostaglandin synthesis through NFKB activation [5], results in the complete loss of embryos when given on days 11 to 16 of pregnancy in the pig [6]. While elevated PTGS2 mediated prostaglandin production by the endometrium of cyclic and pregnant gilts could be mediated by mechanisms independent of NFKB, results suggest a relationship between the NFKB and elevated PTGS2.

We hypothesize that NFKB activation of PTGS2 transcription in the uterine endometrium is in part driven [4] by progesterone, as *PTGS2 *expression in the luminal epithelium is present during the estrous cycle without conceptus secretion of IL1B [3] and estradiol-17β [7]. Plasma concentrations of progesterone are high during diestrus and cause a specific down-regulation of progesterone receptor (PGR) protein in the luminal epithelium of pigs on days 10 to 12 of the estrous cycle and pregnancy [8]. The down-regulation of PGR in the luminal epithelium during the peri-implantation period is associated with changes in endometrial gene expression that lead to either uterine receptivity for conceptus development and attachment or to pathways that release prostaglandin F2α to regress the corpora lutea and initiate a return to estrus [9]. In fact, the association between PGR down-regulation and the opening of the implantation window is not limited to the pig as PGR down-regulation in the uterine luminal epithelium occurs prior to implantation in humans [10,11], baboons [12], sheep [13], cattle [14] and horses [15].

Although the specific down-regulation of PGR in the luminal epithelium has been well established, the pathway for this important biological event is not known. Progesterone receptor is capable of regulating the transcription of NFKB induced genes as RELA and PGR are mutually repressive of each other [16]. Thus, we propose that PGR expression in the uterine LE is regulated by interactions with NFKB. The objective of this study was to characterize the contributing factors to the activation of NFKB1 and RELA during the estrous cycle and early pregnancy in the endometrium of the pig.

## Methods

### Animals

Research was conducted in accordance with the Guiding Principles for Care and Animals use for this project was approved by the Oklahoma State Institutional Animal Care and Use Committee. Cyclic, crossbred gilts of similar age (8 to10 mo) and body weight (100 to 130 kg) were observed for estrous behavior twice daily in the presence of an intact boar. The onset of estrus was designated day 0 of the estrous cycle. Gilts were mated naturally with fertile boars at the onset of their second estrus (day 0 of estrous cycle) and again 24 h later.

### Tissue collection

Once observed in estrus, gilts were randomly assigned to be either pregnant or cyclic. Gilts destined for pregnancy were mated by natural service from intact boars at the onset of estrus and again 24 h later. Uteri were collected from cyclic gilts on days 0, 5, 7.5, 10, 12, 13, 15 and 17 of the estrous cycle whereas uteri were collected from pregnant gilts on days 10, 12, 13, 15 and 17. These days were utilized as they are representative of hormonal changes that occur during each phase of the estrous cycle and allow for the analysis of the confounding impact conceptuses may contribute during pregnancy. Gilts were hysterectomized (n = 4 gilts/status/day) by performing a midventral laporatomy as previously described [17]. Following induction of anesthesia with 1.8 mL of i.m. administration of a cocktail consisting of 2.5 mL Xylazine (100 mg/mL) and 2.5 mL Vetamine (Ketamine HCL; 100 mg/mL) in 500 mg of Telazol (Tiletamine HCl and Zolazepum HCl), anesthesia was maintained with a closed circuit system of halothane (5%) and oxygen (1.5 liters/min). Immediately following removal, each uterine horn from both cyclic and pregnant gilts was flushed with 20 mL of a physiological saline and conceptuses were removed. Following flushing, one uterine horn was cut along its anti-mesometrial border and endometrial samples representing several regions of the uterine horn (5 to10 g) was removed with sterile scissors and snap-frozen in liquid nitrogen and stored at -80°C until analysis. Cross sections of uteri were also fixed in 4% paraformaldehyde and dehydrated in 70% ethanol to use for *in situ *hybridization.

### RNA isolation

Total RNA was extracted from uterine endometrium by using the RNAwiz reagent (Ambion, Inc., Austin, TX) according to manufacturer's recommendations. Approximately 500 mg of endometrium was homogenized in 5 mL RNAwiz reagent using a Virtishear homogenizer (Virtis Company Inc., Gardiner, NY). The RNA pellets were rehydrated in nuclease-free H_2_O and stored at -80°C. The RNA content was estimated spectrophotometrically and purity estimated using the 260:280 ratio. RNA quality and integrity was assessed by using gel electrophoresis and no differences between samples were noticed.

### Protein extraction

Cytoplasmic and nuclear protein fractions were prepared using a previously established method [18,19]. Briefly, 5.0 mg of endometrium from cyclic (days 5, 10 and 13) and pregnant (days 10 and 13) gilts (n = 4/day/status) was pulverized in liquid nitrogen. Pulverized tissue was re-suspended in lysis buffer [150 mM NaCl, 10 mM Hepes-KOH (pH 7.9), 1 mM ethylenediaminetetraacetic acid (EDTA), 0.6% NP-40 and 1× Protease inhibitor cocktail (Pierce Biotechnology, Rockford, IL)] and homogenized with a loosely fitted pestle in a Dounce homogenizer (Wheaton, Millville, NJ). The homogenate was centrifuged at 1000 × g for 1 min at 4°C, and then the supernatant was transferred to a new tube, incubated in ice for 5 min followed by additional centrifugation at 3300 × g, for 5 min at 4°C. The supernatant, containing the cytoplasmic protein fraction was transferred and stored at -80°C while the pelleted nuclei were resuspended in 200 μl of nuclear extraction buffer [25% glycerol, 20 mM Hepes KOH (pH 7.9), 420 mM NaCl, 1.2 mM MgCl_2_, 0.2 mM EDTA, 0.5 mM DL-Dithiothreitol (DTT) and 1× protease inhibitor cocktail]. Resuspended nuclei were incubated on ice for 30 min and vortexed intermittently. The nuclear suspension was centrifuged at 12 000 × g for 5 min at 4°C. Following centrifugation, the supernatant containing the nuclear protein extract was transferred to a new tube and stored at -80°C. Total protein concentrations were determined using the method of Bradford [20] through the Bio-Rad protein assay (Bio-Rad Laboratories, Hercules, CA).

### RELA ELISA

The presence of the NFKB subunit RELA in nuclear and cytoplasmic protein extractions was determined using the TransAM NFKB RELA transcription factor assay kit (Active Motif, Carlsbad, CA). The assay uses a 96-well format for which each well contains immobilized oligonucleotide containing the NFKB consensus sequence. NFKB RELA was assayed in both nuclear and cytoplasmic protein fractions collected from endometrium of gilts on days 5, 10 and 13 of the estrous cycle and days 10 and 13 of pregnancy (n = 4 gilts per status/day). All samples, positive control nuclear extract, and blank (using nuclear extraction buffer) were assayed in duplicate according to the manufacturer's recommendations. Positive control nuclear extract was included in the kit and excess wild-type and mutated NFKB consensus sequence oligo was added in excess to other control wells to determine specificity. Following protein/DNA binding and washing of the wells, anti-RELA primary and HRP-conjugated secondary antibodies, included with the assay kit, were used to determine relative amounts of bound RELA. Following secondary antibody binding, the wells were developed colorimetrically and absorbance at 450 nm was determined. The optical density (OD) for each sample was first corrected by subtraction of the OD of the blank and subsequently corrected for total protein in the sample.

### Histological localization of RELA

Immunohistochemistry was conducted using a rabbit anti-human polyclonal antibody for the RELA subunit of NFKB (sc-372; Santa Cruz Biotechnology, Santa Cruz, CA) as primary antibody at a working concentration of 2 μg/mL. Donkey anti-rabbit secondary antibody containing a fluorescent Cy2 conjugate (711-225-152, Jackson ImmunoResearch Laboratories, West Grove, PA) was used for fluorescent detection. Negative control slides were also developed where a substituting non-immune serum for the primary antibody. Tissue sections were then scored for nuclear localization of NFKB in uterine luminal epithelial (LE) cells by three individuals who were blinded to pregnancy status and day. The scoring was based upon nuclear fluorescent intensity and done independently by the three individuals using a 0 to 5 scale where 0 indicated no nuclear localization and 5 indicated high nuclear localization.

### *In situ *hybridization

The location of progesterone receptor (PGR), estrogen receptor 1 (*ESR1*, also known as estrogen receptor α), and receptor activator of NFKB (*TNFRSF11A*, also known as Receptor Activator of NFKB (*RANK*)) mRNA was determined in porcine uterine cross-sections by radioactive *in situ *hybridization using methods previously described [21]. Paraffin-embedded cross-sections (~5 μm) were deparaffinized, rehydrated, and deproteinated; then hybridized with radiolabeled antisense or sense porcine cRNA probes (5.0 × 10^6 ^counts per minute/slide) synthesized through *in vitro *transcription with [α-^35^S] uridine 5-triphosphate (MP Biomedicals, Irvine, CA) using a linearized plasmid template. Following the hybridization washes, and RNase A digestion, hybridized slides were exposed to Biomax maximum resolution film (Eastman Kodak, New Haven, CT) overnight to determine signal strength. Autoradiography was performed using NTB liquid photographic emulsion (Eastman Kodak). Slides were dipped in emulsion and exposed at 4°C based on the strength of signal from the Biomax film (2 weeks for PGR and ESR1, and 8 weeks for TNFRSF11A), developed in Kodak D-19 developer, counterstained with Harris modified hematoxylin (Fisher Scientific, Fairlawn, NJ), dehydrated, and protected with cover slips.

Digital photomicrographs of *in situ *hybridization, brightfield and darkfield images of liquid emulsion autoradiography, were collected using a Nikon Eclipse E6000 microscope interfaced with the CoolSNAPcf digital camera equipped with a cooled charge-coupled device (Photometrics, Tucson, AZ) and imaging software (MetaVue, Molecular Devices, Downington, PA).

### Quantitative one-step RT-PCR

Quantitative analysis of RELA, NFKB1, TNFRSF11A, Toll-like receptor-4 (TLR4), and inhibitors of κB (NFKBIA and NFKBIB) and were assayed using quantitative real-time RT-PCR and a fluorescent reporter as previously described [4]. PCR amplification was conducted using the ABI PRISM 7500 Sequence Detection System (PE Applied Biosystems, Foster City, CA). Real-time detection during each amplification cycle was done by using a sequence specific dual-labeled fluorescent probe designed to have a 5' reporter dye (6-FAM) and a 3' quenching dye (TAMRA) nested between the forward and reverse sequence specific primers. All primers and probes utilized for quantitative analysis for each target gene are presented in Table 1. PCR reactions were conducted using the qRT-PCR kit (Eurogentec North America, San Diego, CA). One hundred nanograms of RNA were assayed for each sample in duplicate. Thermal cycling conditions using the dual labeled probe were 50°C for 30 min, 95°C for 15 min, followed by 40 repetitive cycles of 95°C for 15 sec and a combined annealing/extension stage, 59°C for 1 min. Fluorescent data acquisition was done during the annealing/extension phase. Based on previous experiments for our lab, 18S ribosomal RNA was assayed as a normalization control to correct for loading discrepancies [3] using a commercially available primer set (Eurogentec North America, San Diego, CA). Following RT-PCR, quantitation of gene amplification was made by determining the cycle threshold (C_T_) based on the fluorescence detected within the geometric region of the semilog view of the amplification plot. Relative quantitative analysis of target gene expression was evaluated using the comparative C_T _method as previously described [4,22]. The ΔC_T _value was determined by subtracting the target C_T _of each sample from its respective ribosomal 18S C_T _value. Calculation of ΔΔC_T _involves using the single greatest sample ΔC_T _value (the sample with the lowest expression) as an arbitrary constant to subtract from all other sample ΔC_T _values. Relative mRNA units for each sample were calculated assuming an amplification efficiency of 2 during the geometric region of amplification, and applying the equation, 2^ΔΔCt^. Relative mRNA units are presented as mean ± SEM.

**Table 1:** Primer and probe sequences used for quantitative RT-PCR analysis

Target^a^	Forward/Reverse Primers (5' → 3')^b^	Fluorescent Reporter^c^	Length of Amplicon^d^	GenBank Accession #^e^
*TNFRSF11A*	GCTGACTCTGGAAGAGAAGGTGTT	ATGTGCTGTCCAGACGGTGGTGGTGCCTGT	192 bp	CB475057
	GCCCTGTCCACATATTCGTCTTCTGT			
*TLR4*	ATGGCCTTTCTCTCCTGCCTGA	ATCTGAGAGCTGGGACCCTTGCGTGCAGGT	139 bp	AB188301
	AGGTCCAGTATCTTGACTGATGTGGG			
*NFKB1*	CCCATGTAGACAGCACCACCTATGAT	ACCAGGCTGGCAGCTCTCCTCAAAGCAGCA	132 bp	NM_001048232
	ACAGAGGCTCAAAGTTCTCCACCA			
*RELA*	ACATGGACTTCTCAGCCCTTCTGA	ACACCTGCTCTGCCCAGAGCACTGGGTT	168 bp	CN155798
	CCGAAGACATCACCCAAAGATGCT			
*NFKBIA*	TGTGATCCTGAGCTCCGAGACTTT	TCTACACCTTGCCTGTGAGCAGGGCTGCCT	143 bp	NM_001005150
	TTGTAGTTGGTGGCCTGCAGAATG			
*NFKBIB*	TCATTCTGCAGGTCCAGGTACTCA	TGGATTTCCTCCTGGGCTTTGCTGCTGGCA	89 bp	AK231853
	CACTTGGCGGTGATTCATCAGCAT			

^a^The amplification target: *TNFRSF11A*, Receptor activator of NFKB; *TLR4*, Toll-like receptor 4; *NFKB1*; RELA, *NFKBIA *and *NFKBIB*, Inhibitors of κB α and β, respectively.

^b^The forward and reverse DNA oligos used in the amplification of the target. Forward and reverse do not necessarily indicate the *in vivo *direction of transcription.

^c^The DNA oligo sequence of the dual-labeled probe (possessing the FAM reporting dye on the 5' end and the TAMRA quenching dye on the 3' end) used to measure amount of amplified target during each cycle of quantitative RT-PCR.

^d^The length of the amplicon created during PCR.

^e^The accession number to the porcine sequence that was utilized during primer and probe design.

### Statistical analysis

Statistical analysis was conducted to determine differences for the RELA assay of endometrial extract, histological determination for RELA nuclear translocation, and the quantitative RT-PCR results. For RELA assay using endometrial extract, the analysis was conducted using the corrected OD values through PROC MIXED of the Statistical Analysis System and was conducted to determine the probably that day, pregnancy status, and protein fraction; as well as all possible interactions affected the observations. Data are presented as mean ± SEM. For nuclear translocation, the average nuclear localization score for the three evaluators was calculated and subjected analyzed by using PROC MIXED (SAS). The statistical model included the effects of day, pregnancy status, and the day by pregnancy status interaction. Quantitative RT-PCR ΔC_T _values; representing the raw, normalized data, were used for analyses, also through PROC MIXED. Analysis of endometrial gene expression tested for the effect of day and status; and day × pregnancy status interactions to determine if the presence of conceptuses influences changes that are occurring across different days. Significance between means of specific effects (*P *< 0.05) was determined by probability differences of least squares means. Figures representing relative mRNA units have superscripts above bars depicting significant differences as determined by the ΔC_T _values (*P *< 0.05).

## Results

### RELA ELISA

**Figure 1:**
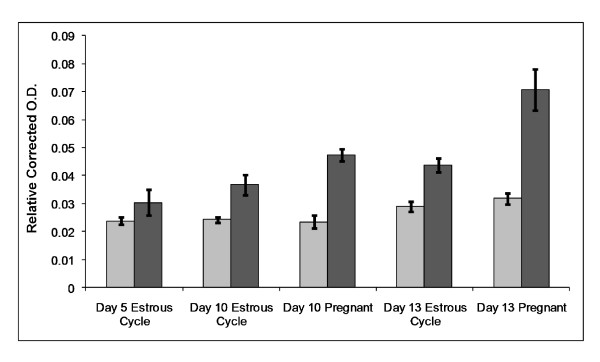
**Transcription factor assay to determine relative amounts of activated RELA**. Nuclear (light bars; day effect, *P *= 0.005) and cytoplasmic (dark bars; day effect, *P *= 0.006; status effect, *P *< 0.001) protein fractions of endometrium collected from gilts on days 5, 10 and 13 of the estrous cycle and days 10 and 13 of pregnancy (n = 4/day/status). Relative optical density for each sample was corrected for total protein concentration in the sample. Data is presented as relative O.D. mean ± SEM.

A day × status (*P *= 0.05) and status × protein fraction (*P *= 0.001) interaction was detected for RELA protein expression. No difference in the amount of RELA in the nuclear fraction of the endometria was observed between the days of the estrous cycle and pregnancy (Figure 1). However, the amount of RELA in the cytoplasmic compartment was greater during the mid-luteal phase (D13) compared to day 5. Furthermore, the amount of RELA in the cytoplasmic fraction was greater than the amount in the nuclear fraction on D10 and D13 in comparison to D5, for which there was no detectable difference in RELA between the cytoplasmic and nuclear protein fractions. While this difference in cellular compartment was present in both cyclic and pregnant gilts, the difference appeared to be greater in the pregnant gilts (Figure 1).

### Immunohistological localization of RELA

**Figure 2:**
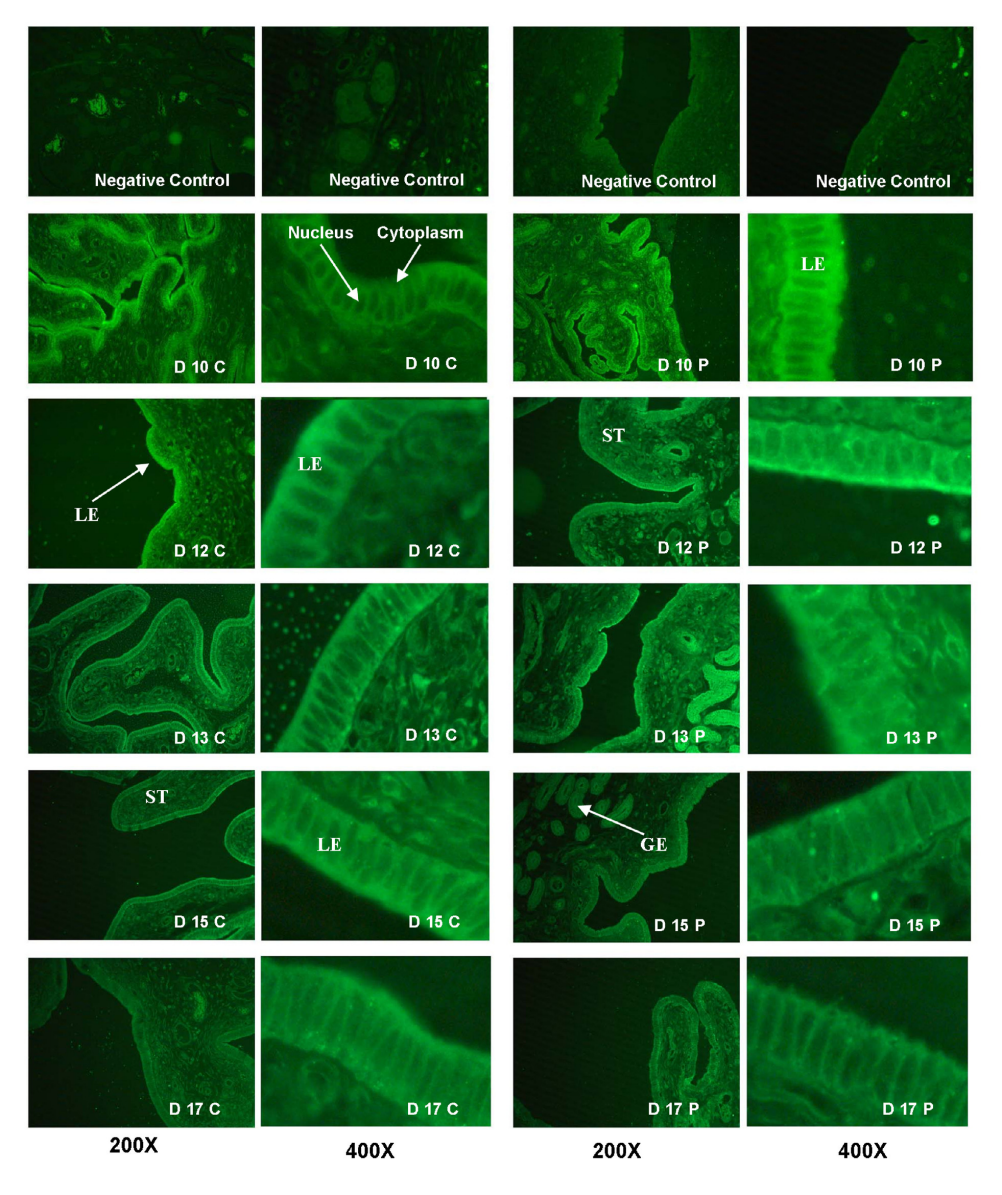
**Immunolocalization of RELA in uterine endometrium**. Endometrium from days 10, 12, 13, 15 and 17 of the estrous cycle (C) and days 10, 12, 13, 15, and 17 of pregnancy (P) were immunostained for RELA. Images are representative from four biological replications. A representative section from d 15 of pregnancy immunolocalized without either the primary or secondary antibody served as negative controls.

**Figure 3:**
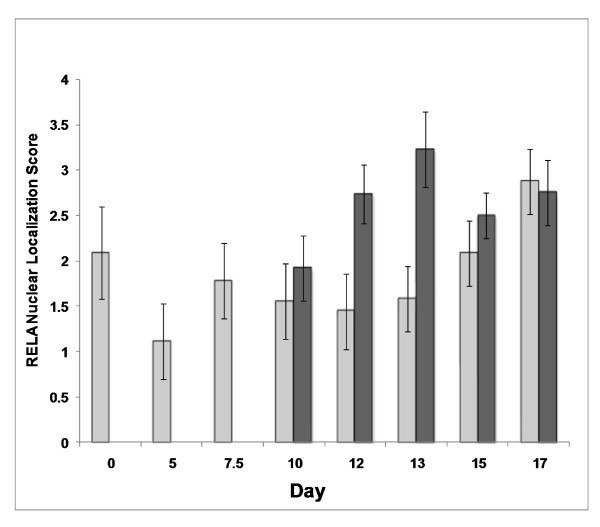
**Translocation of RELA in the luminal epithelial cells**. Nuclear translocation was visually determined and assigned a score; 0 being no localization within the nucleus and 5 being significant localization within the nucleus. Translocation of an activated NFKB heterodimer, as determined through RELA antibody binding, was affected by pregnancy status (*P *= 0.004) but not by day (*P *= 0.11) or day × status interaction (*P *= 0.13). Dark bars represent days of pregnancy and light bars represent days of the estrous cycle. Data are presented as mean score ± SEM.

NFKB RELA was localized in the nucleus of uterine luminal epithelial cells during all days of the estrous cycle and early pregnancy (Figure 2). In addition, RELA was also localized in the underlying stromal cells and the glandular epithelium in most samples and was highly detectable on days 13 and 15 of pregnancy. Translocation of the transcription factor into the nucleus of luminal epithelial cells was affected by pregnancy status (*P *= 0.004), but not by day (*P *= 0.11) or a day × status interaction (*P *= 0.13) (Figure 3). The greatest amount of RELA translocated to the nucleus was on D12 and D13 of pregnancy, during the opening of the implantation window.

### *In situ *hybridization

#### Estrogen receptor 1

**Figure 4:**
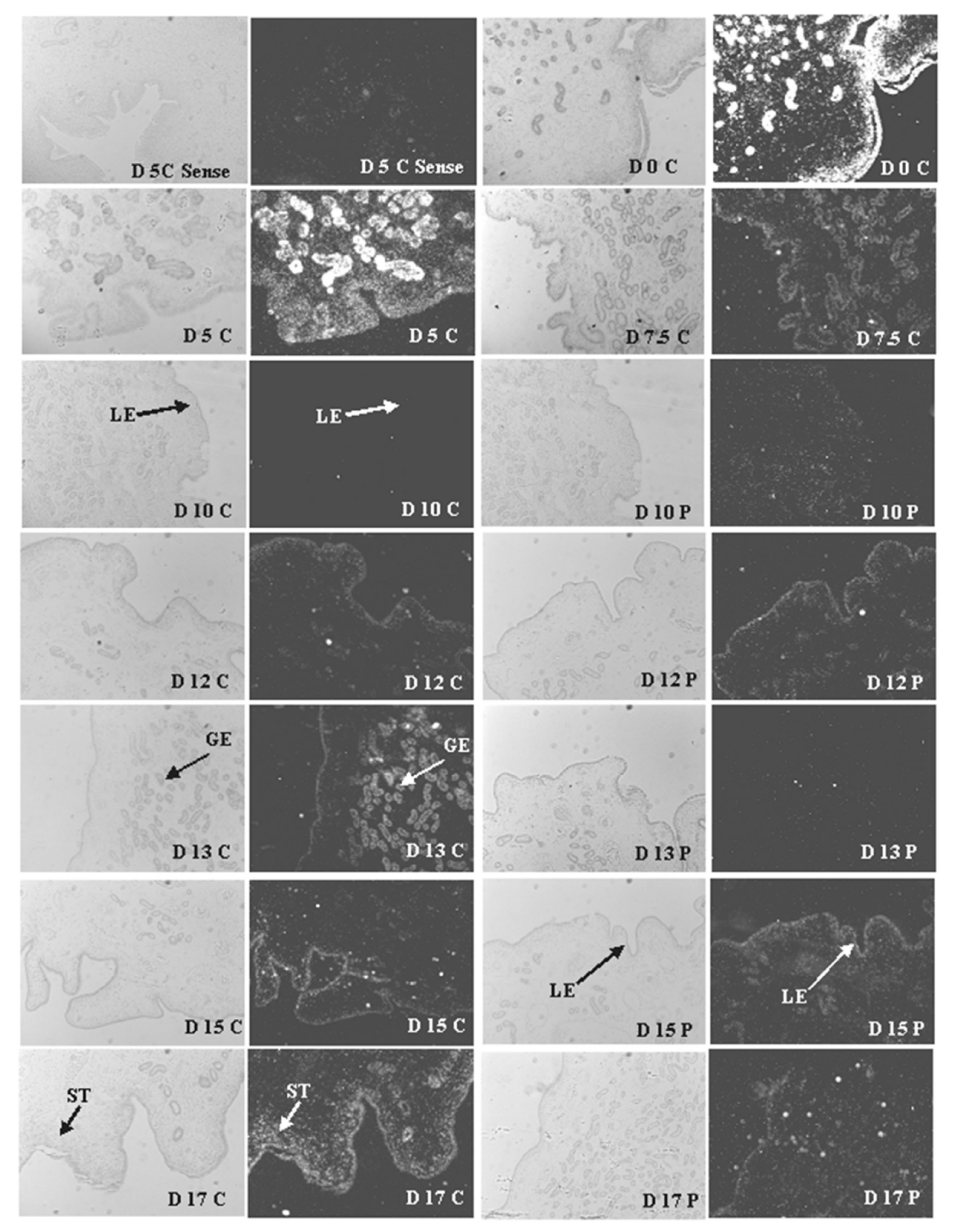
***In Situ *hybridization analysis of *ESR1 *mRNA expression in porcine endometrium**. Protected transcripts in endometrium from days 0, 5, 7.5, 10, 12, 13, 15 and 17 of the estrous cycle and days 10, 12, 13, 15 and 17 of pregnancy were visualized by liquid emulsion autoradiography and imaged under bright-field and dark-field illumination. Note the greatest expression is prior to (day 17), during (day 0) and after estrus (day 5). *ESR1 *expression is abundant in the luminal epithelium (LE) and glandular epithelium (GE) during days 0 and 5 while only in the LE on day 17 of the estrous cycle. A representative day 5 section was hybridized with radiolabeled sense cRNA probe to serve as a negative control. All other images are representative from four biological replications. 4× Objective and 10× eyepiece for original magnification 40×.

Endometrial *ESR1 *mRNA was most abundant in the uterine luminal epithelia (LE), glandular epithelia (GE) and stroma (ST) at estrus (day 0) (Figure 4). By D5, expression was still apparent in all cell types of the endometrium albeit at a reduced abundance. Decline in *ESR1 *abundance continued in all cell types being nearly devoid by D7.5 of the estrous cycle and D10 of both the estrous cycle and pregnancy. On D12 of pregnancy, *ESR1 *mRNA abundance was elevated in the LE. Expression during D12 of the estrous cycle was modest compared to D13 cyclic endometrium exhibiting elevated *ESR1 *mRNA in the GE. In the GE, *ESR1 *mRNA was transient as expression in cyclic gilts steadily increased in the LE on D15 and D17 of the estrous cycle. While *ESR1 *expression on D15 of was similar between cyclic and pregnant gilts, by day 17 LE expression was much less during pregnancy (Figure 4).

#### Progesterone receptor

**Figure 5:**
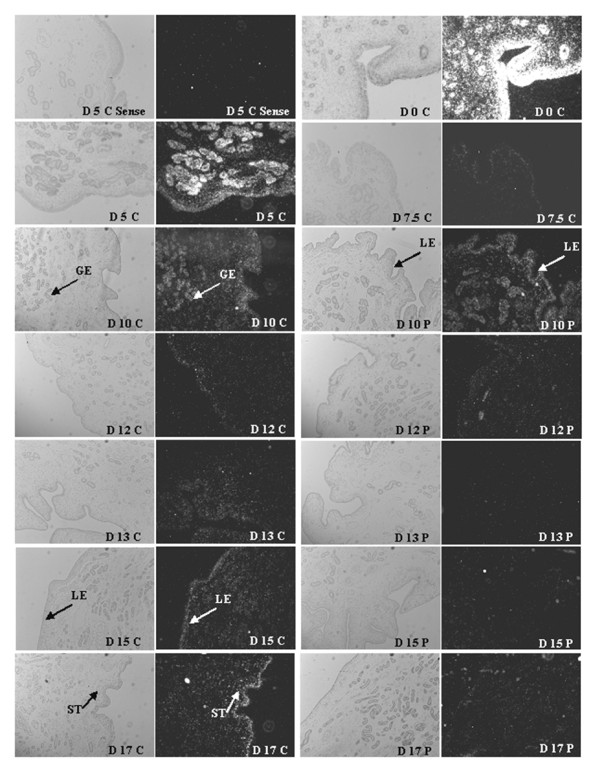
***In Situ *hybridization analysis of *PGR *mRNA expression in porcine endometrium**. Protected transcripts in endometrium from days 0, 5, 7.5, 10, 12, 13, 15 and 17 of the estrous cycle and days 10, 12, 13, 15 and 17 of pregnancy were visualized by liquid emulsion autoradiography and imaged under bright-field and dark-field illumination. Note the greatest expression is during (day 0) and after estrus (day 5) abundant in the luminal epithelium (LE) and glandular epithelium (GE) and stromal (ST) cell types. *PGR *expression is greatly reduced in the LE and GE by day 10 and nearly devoid on day 12 of the estrous cycle and pregnancy, although still present in ST cells. A representative day 5 section was hybridized with radiolabeled sense cRNA probe (sense) to serve as a negative control. All other images are representative from four biological replications. 4× Objective and 10× eyepiece for original magnification 40×.

Similar to *ESR1 *mRNA, *PGR *mRNA was present in the LE, GE and ST at estrus (D0) and was substantially lower thereafter (Figure 5). *PGR *mRNA on D7.5 and D10 of the estrous cycle was detectable, although greatly reduced, while it was near devoid in the uterine epithelium by D12 of the estrous cycle and early pregnancy except for expression persisting in the ST cells. *PGR *expression remained at the lowest level throughout the remainder of the estrous cycle and pregnancy until its expression began to increase in the LE of cyclic gilts following luteolysis while remaining very low to undetectable in the LE at D15 and D17 in pregnant gilts (Figure 5).

#### Receptor activator of NFKB

**Figure 6:**
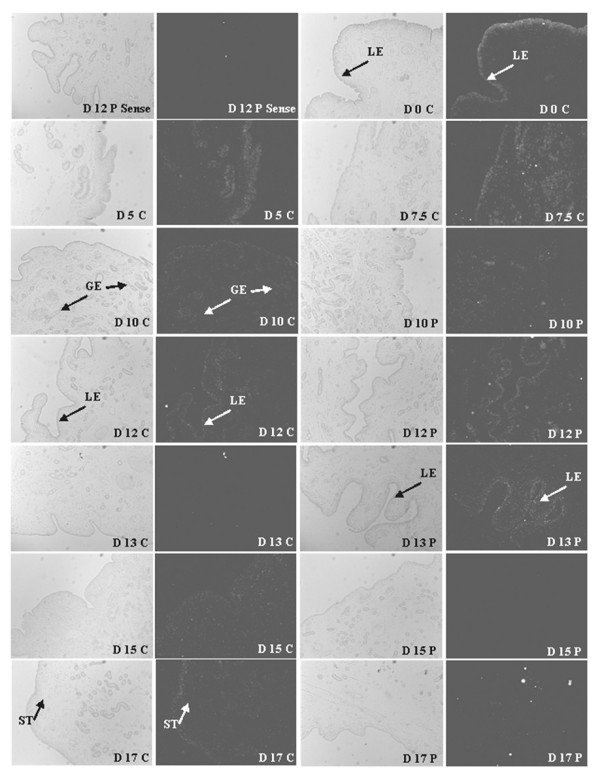
***In Situ *hybridization analysis of receptor activator of NFKB (*TNFRSF11A*)**. Protected transcripts in endometrium from days 0, 5, 7.5, 10, 12, 13, 15 and 17 of the estrous cycle and days 10, 12, 13, 15 and 17 of pregnancy were visualized by liquid emulsion autoradiography and imaged under bright-field and dark-field illumination. While expression was low in all tissues evaluated, evidence of *TNFRSF11A *expression is detected in the LE on day 12 of the estrous cycle and pregnancy. A representative day 12 pregnant section was hybridized with radiolabeled sense cRNA probe (sense) to serve as a negative control. All other images are representative from four biological replications. 4× objective and 10× eyepiece for original magnification 40×.

*TNFRSF11A *expression in the uterus was undetectable for many of the days of the estrous cycle and pregnancy although the greatest expression appeared to occur in LE on D0 during estrus. The expression was visible, although modestly, in the LE and ST during the mid-luteal phase, D10 to D13 of both cyclic and pregnant gilts (Figure 6). While LE expression of *TNFRSF11A *was low on D15 and D17 of the estrous cycle, LE expression in pregnant gilts was devoid on the same days (Figure 6).

### Quantitative RT-PCR

We conducted quantitative RT-PCR to determine expression changes of *TNFRSF11A *and *TLR4 *(receptors capable of inducing NFKB activity) and two genes that encode subunits for NFKB (*RELA *and *NFKB1*).

#### NFKB mediating receptors

**Figure 7:**
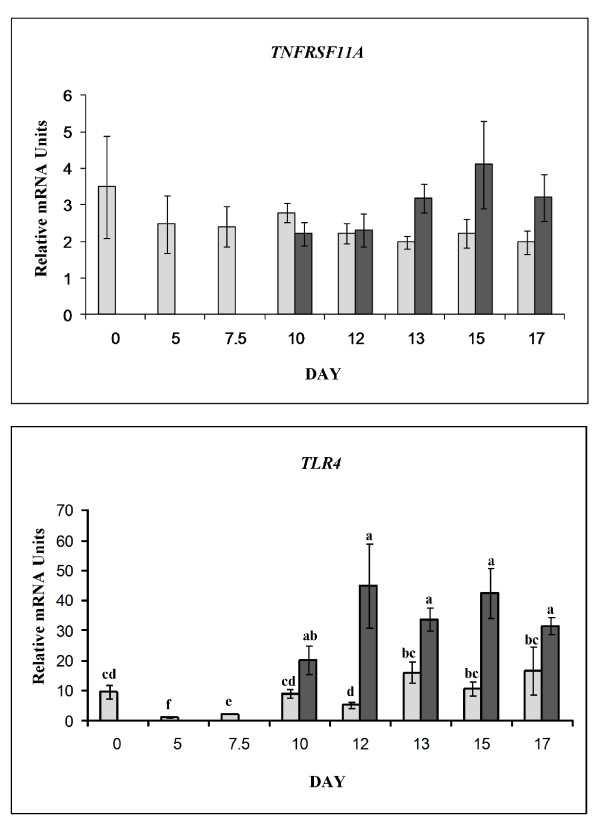
**Relative mRNA abundance for receptor mediators of NFKB activation**. Expression differences for endometrial *TNFRSF11A *(Top panel; effect of day, *P *< 0.93; effect of status, *P *= 0.34) and *TLR4 *(Lower panel; day × status effect, *P *= 0.017), in cyclic (light bars) and pregnant (dark bars) gilts. Relative abundance of mRNA was calculated from the quantitative RT-PCR analysis as described in *Materials and Methods*. Bars without common lowercase superscripts represent a statistical difference (*P *< 0.05) between day/status combinations. Superscripts are not included in the upper panel as no main or interacting effects were detected. Relative mRNA units are presented as mean ± SEM.

There was no significant day (*P *= 0.92) or status (*P *= 0.34) effect detected for the expression of *TNFRSF11A*. Messenger RNA levels were not different in uterine endometrium throughout the days of the estrous cycle and early pregnancy in pigs (Figure 7).

There was a day × status interaction (*P *= 0.017) on the mRNA abundance for *TLR4 *(Figure 7). Expression was lowest on D5 of the estrous cycle and greatest on D13 of pregnancy. While expression on D10 through D17 of the estrous cycle were greater than D5 and D7.5 (*P *< 0.05), endometrium of pregnant gilts expressed significantly more *TLR4 *mRNA transcript than cyclic gilts on D10, D12, D13, D15 and D17 (*P *< 0.05).

#### NFKB subunits: NFKB1 and RELA

**Figure 8:**
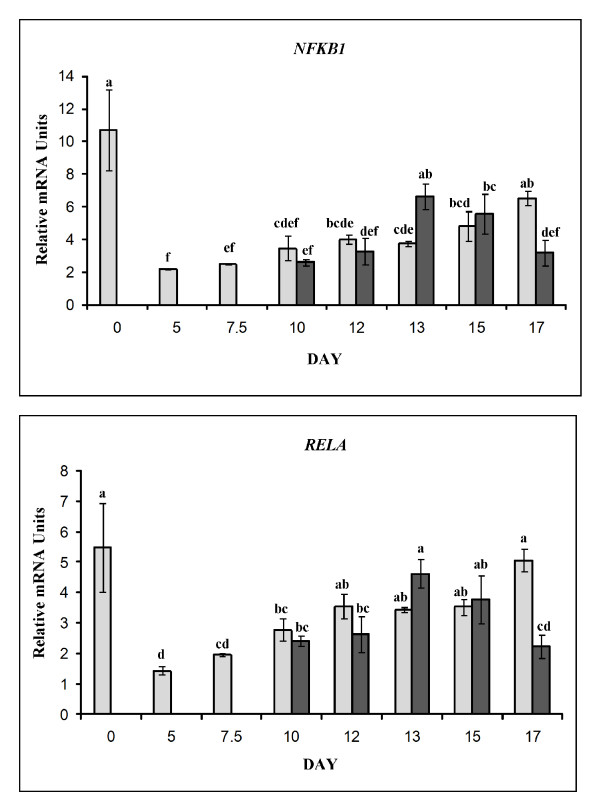
**Relative mRNA abundance for NFKB subunits**. Expression differences for endometrial *NFKB1 *(Top panel; day × status interaction, *P *< 0.01) and *RELA *(Lower panel; day × status effect, *P *= 0.017), in cyclic (light bars) and pregnant (dark bars) gilts. Relative abundance of mRNA was calculated from the quantitative RT-PCR analysis as described in *Materials and Methods*. Bars without common lowercase superscripts represent a statistical difference (*P *< 0.05) between day/status combinations. Relative mRNA units are presented as mean ± SEM.

A significant day × pregnancy status interaction was detected for *NFKB1 *gene expression (*P *< 0.01). Messenger RNA abundance for *NFKB1 *was greatest during estrus and least on day 5 (Figure 8). *NFKB1 *expression increased steadily during diestrus being 2 to 3-fold greater in endometrium collected from D12, D13, D15 and D17 compared to D5 (*P *< 0.05). On D13 of gestation, expression was about 2-fold greater when compared to D13 of the estrous cycle (*P *< 0.05) while the inverse was true when comparing D17 of the estrous cycle and pregnancy (Figure 8).

Similar to *NFKB1*, a day × status interaction (*P *< 0.013) affected the mRNA abundance of *RELA *in the endometrium from cycle and pregnant gilts. Like the *NFKB1 *subunit, *RELA *expression was greatest in endometrium collected during estrus, about 4-fold greater than D5 of the estrous cycle which was the lowest expression of *RELA *mRNA. After D5 of the estrous cycle, expression increased in both cyclic and pregnant gilts on D10, D12, D13, and D15 (*P *< 0.05; Figure 8). While expression remained elevated on D17 of the estrous cycle, expression returned to basal levels on D17 of gestation, representing about a 2.3-fold difference in gene expression (*P *< 0.05).

#### Inhibitors of NFKB

**Figure 9:**
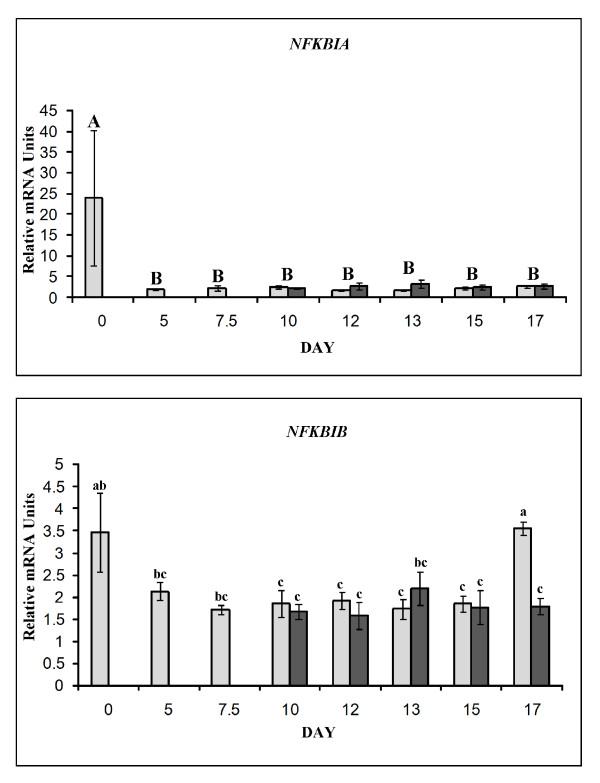
**Relative mRNA abundance for endometrial inhibitors of NFKB activation**. *NFKBIA *(Top panel; effect of day, *P *< 0.001) and *NFKBIB *(Lower panel; day × status effect, *P *= 0.079), in cyclic (light bars) and pregnant (dark bars) gilts. Relative abundance of mRNA was calculated from the quantitative RT-PCR analysis as described in *Materials and Methods*. Bars without common lowercase superscripts represent a statistical difference (*P *< 0.05) between day/status combinations whereas differences in uppercase superscripts represent statistical differences between days of gestation. Relative mRNA units are presented as mean ± SEM.

Gene expression for *NFKBIA *was affected by day (*P *= 0.001) but not status (*P *= 0.15). Status did not affect the expression of *NFKBIA *(*P *= 0.23). *NFKBIA *mRNA abundance was greatest during estrus, being at least 7-fold greater in comparison to all other days assayed (*P *< 0.05). There were no differences in gene expression between all other days as mRNA abundance remained at a consistent level from days 5 through 17 (Figure 9).

There was a tendency for a day × status interaction (*P *= 0.08), day (*P *= 0.07) and status (*P *= 0.09) for *NFKBIB *gene expression. The greatest expression differences existed during estrus and day 17 of estrous cycle. *NFKBIB *mRNA abundance was greater (*P *< 0.05) in endometrium of day 17 cyclic gilts with expression levels similar to day 0 but was about 2-fold greater compared to all other days during diestrus and early pregnancy (days 5 to 15). No differences were detected between days 5 to 15 of the estrous cycle or pregnancy (Figure 9).

## Discussion

The establishment of pregnancy requires diversion from the pathway of cyclicity and return to estrus following the opening of the implantation window and uterine receptivity in pigs. Progesterone-driven transcriptional regulation of specific endometrial factors during the mid-luteal phase contributes to both induction of luteolysis resulting in return to estrus in cyclic animals and conceptus growth and expansion to establish pregnancy in mated females. We have suggested a relationship between PGR expression and the activation of NFKB in the endometrial LE regulates the transcription of specific genes such as PTGS2 [4,9].

Establishment of pregnancy in mice has been shown to involve the NFKB system. Not only does the activation of NFKB during the implantation window in mice occur [19]; it has also been shown to be required for uterine receptivity through its affects on leukaemia inhibitory factor (LIF) expression, as LIF administration restores normal implantation during pregnancy when NFKB function is compromised [23]. Interestingly, peak LIF secretion by the pig uterus occurs on day 12 of the estrous cycle [24], as well as up-regulation of uterine PTGS2 [4], an NFKB regulated gene [5,25], and we now demonstrate an increased *NFKB1 *and *RELA *gene expression, both of which can be induced through NFKB [26]. These NFKB regulated factors are all in congruence with the opening of the implantation window in the pig. Based on the expression patterns of these genes in conjunction with the RELA ELISA it appears that transcriptional control of uterine endometrium during the initiation of implantation is regulated by NFKB possessing the RELA subunit, and that during pregnancy, NFKB is more actively translocated into the nucleus in the luminal epithelium.

Steroid hormones, as well as their receptors, have in many studies been shown to affect NFKB activity in a variety of tissues. Kalkhoven et al. [16] demonstrated the ability of both PGR and NFKB RELA to be mutually repressive of each other, which suggests that the loss of PGR during the opening of the implantation window may be related to elevated expression of NFKB regulated genes in the uterine endometrium during the estrous cycle and early pregnancy. Loss of PGR expression in the uterine LE is temporally associated with uterine receptivity in the pig and other numerous mammalian species [10-15]. The ability for PGR down-regulation to affect the timing of uterine receptivity appears to carry over into the timing of the overall length of estrous cycle.

The hypothesis that progesterone induced loss of PGR regulates uterine receptivity and the estrous cycle is supported by the demonstration of shortened estrous cycles in sheep and cattle given progesterone supplementation shortly after estrus, prior to normal endogenous progesterone secretion by the corpora lutea [27,28]. Additionally, administration of the PGR antagonist, mifepristone, on days 3-5 of the ovine estrous cycle results in a delay of luteolysis [29]. Collectively, these studies suggest that PGR is involved in the regulation of prostaglandin (PG) production. Indomethacin, an inhibitor of NFKB induced PTGS2 expression [5], when given to pigs during early gestation ablates PG production [6], suggesting that uterine PG production during diestrus in the pig is NFKB regulated. Demonstrating that altering either PGR expression levels or NFKB activation, these studies independently suggest the ability of both PGR and NFKB to regulate PG production during the period of luteolysis.

The temporal affiliation between the gene expression patterns of TLR4 and TNFRSF11A and NFKB inducible genes; NFKB1, RELA, PTGS2 [4] and LIF [24] in the uterine LE is suggestive of the involvement of TLR4 and/or TNFRSF11A during the activation of NFKB during the initiation of uterine receptivity during the estrous cycle and early pregnancy in pigs. Because a mutual repression between RELA and PGR exists [16], what regulates this activation of NFKB and potentially, the subsequent down-regulation of PGR in the uterine endometrium remains unclear. The expression of TNFRSF11A and its ligand (TNSF11), both involved in bone remodeling and mammary gland development [30,31], provide one such potential mechanism. Expression of TNSF11 can be stimulated by progesterone [32] suggesting the potential of TNFRSF11A mediated NFKB activation in the pig endometrium if TNSF11 is capable of signaling through endometrial stromal cells which still express PGR during the implantation window in the pig [8]. This novel hypothesis has been presented [9] and is supported by the expression of *TNFRSF11A *in the uterine endometrium; however no communications to our knowledge have indicated the presence of TNFRSF11A or its ligand in the uterus of other mammalian species.

TLR4 also provides a likely mechanism of NFKB activation in the pig as its expression pattern is temporally associated with that of the NFKB regulated genes, *PTGS2 *[4], *LIF *[24], and the *NFKB1 *and *RELA *in the pig uterine endometrium. TLR4 expression has been demonstrated in endometrial LE of a variety of species. In addition to the pig, TLR4 has also been shown to be expressed in the uterine LE and ST of mice [33], women [34,35] and cattle [36]. While TLR4 was originally described for its ability to respond to the gram-negative cell wall component, lipopolysaccharide (LPS; Poltorak et al., 1998) recent data suggests a variety of endogenous ligands, such as heat shock proteins (HSPs) 60 and 70, fibrinogen, fibronectin, heparan sulfate, certain β-defensins, and high mobility group box 1 protein also possess the ability to stimulate TLR4 signaling [37]. While the suggestion that TLR4 expression in the uterus allows the ability to respond to foreign bacterial pathogens is valid, its roles in uterine biology in conjunction with endogenous ligand expression should not be overlooked. Of the other mentioned endogenous ligands, fibronectin and heparan sulfate have both been suggested to be involved in the attachment of the pig conceptus to the uterine LE [38], occurring temporal and spatially to TLR4 increased expression in the uterine endometrium.

Based on these data and the literature presented hitherto, we hypothesize that NFKB activation is a critical component to the opening of the implantation window in pigs. However, stringent regulation of NFKB activation is necessary to prevent a severe inflammatory response, which could otherwise potentially compromise pregnancy. It is possible that the conceptus could augment the activation of NFKB through at least two mechanisms. The first possible mechanism is the spatiotemporal peak in conceptus secretion of IL1B with uterine expression of IL1 receptors on day 12 of gestation [3]. The second possible mechanism for augmented NFKB activation is through conceptus expression of TLR4 endogenous ligands; fibronectin and fibrinogen [37], both of which are up-regulated 8.9 and 12.8 fold during days 11 to 14 of gestation, respectively [39]. Both of these mechanisms seem plausible as the amount of cytoplasmic RELA sequestered in the cytoplasm was greatest on day 13 of pregnant gilts compared to cyclic gilts. Bovine endometrial explants respond to LPS, the bacterial TLR4 ligand, by increasing PG production, with a notable increased PGE to PGF ratio, while *in vitro *stimulation of LE or ST cells with LPS resulting in *PTGS2 *gene expression could be ablated with an LPS antagonist [36]. Prostaglandin production peaks during the mid-luteal phase for both PGE and PGF during both the estrous cycle and pregnancy in the pig; however, a marked increase in the PGE:PGF ratio is detected in pregnant gilts compared to cyclic gilts [40], further suggesting a relationship between pregnancy PG production ratios and TLR4 conceptus stimulation.

While both the RELA ELISA and RELA immunohistochemistry suggest the presence of RELA in the endometrium it is difficult to accurately identify in *in vivo *samples the precise timing of nuclear translocation as it generally occurs very rapidly and can be tightly regulated. Total endometrial RELA detected was greatest in pregnant gilts supports conceptus augmented NFKB activation, however the majority appears limited to the cytoplasm, indicative of regulatory mechanisms preventing overwhelming inflammatory response. The concomitant estrogen secretion by the conceptus [7] temporal to conceptus production of both IL1B and endogenous TLR4 ligands may be one way NFKB transcriptional activity is regulated. Estrogen, signaling through ESR1 and not ESR2, has the *in vitro *ability to prevent activated NFKB in the cytoplasm from being translocated into the nucleus of the cell and inducing transcription [41]. Interestingly, the up-regulation of ESR1 on days 12 and 13 of gestation in the uterine LE is temporally associated with the elevated uterine luminal estradiol-17β [7] and the pregnancy associated elevation of activated RELA in the cytoplasmic protein fractions. Typically, the degradation of NFKB inhibitors results not only in the ability for the activated NFKB dimer to bind DNA, but also exposes the nuclear localization signal necessary for expedited transport to the nucleus of the cell [2]. These data suggest that while the NFKB1/RELA is capable of binding DNA, the heterodimer remains primarily in the cytoplasm of the cell. The temporal relationship of endometrial ESR1 expression and conceptus estradiol secretion [7,42] may provide a potential mechanism by which this occurs. If this mechanism does occur during pregnancy in the pig, it is likely that other mechanisms are also in place as cytoplasmic RELA also increases in uterine endometrium from gilts during the estrous cycle.

The opening of the implantation window is the initiating point of the period during gestation at which specific endometrial alterations occur to allow the attachment of the conceptus trophectoderm. It is this period of development that requires the formation of a communication network between the developing conceptus and the maternal endometrium with dysfunction on the part of resulting in the inability to establish pregnancy. Steroid hormones, progesterone and estrogen, no doubt serve a fundamental responsibility in the pattern of endometrial secretions and the induction of uterine receptivity. The steroid hormone receptors ESR1 and PGR, through their ability to affect the transcriptional activity of NFKB, are likely a critical component to inducing and regulating the degree of transcription necessary in the uterine endometrium for the establishment of pregnancy in the pig.

## Competing interests

The authors declare that they have no competing interests.

## Authors' contributions

JWR (Ross), MDA and RDG conceived, designed and coordinated much of this project and conducted surgical recovery of uterine tissue samples. Ross conducted the majority of the lab work and MDA contributed. PR conducted TLR4 quantitative PCR analysis. JWR (Ritchey) contributed to sample preparation for the histology presented in the manuscript. DM and ML conducted RELA immunohistochemisty and analysis. KH and TES were responsible for assistance in conducting *in situ *hybridization. Ross prepared the initial draft of the manuscript and all authors contributed to the final version.
